# Oncolytic Effects of a Novel Influenza A Virus Expressing Interleukin-15 from the NS Reading Frame

**DOI:** 10.1371/journal.pone.0036506

**Published:** 2012-05-01

**Authors:** Marijke van Rikxoort, Martin Michaelis, Markus Wolschek, Thomas Muster, Andrej Egorov, Joachim Seipelt, Hans Wilhelm Doerr, Jindrich Cinatl

**Affiliations:** 1 Institut für Medizinische Virologie, Klinikum der Johann Wolfgang Goethe-Universität, Frankfurt am Main, Germany; 2 AVIR Green Hills Biotechnology, Vienna, Austria; McMaster University, Canada

## Abstract

Oncolytic influenza A viruses with deleted NS1 gene (delNS1) replicate selectively in tumour cells with defective interferon response and/or activated Ras/Raf/MEK/ERK signalling pathway. To develop a delNS1 virus with specific immunostimulatory properties, we used an optimised technology to insert the interleukin-15 (IL-15) coding sequence into the viral NS gene segment (delNS1-IL-15). DelNS1 and delNS1-IL-15 exerted similar oncolytic effects. Both viruses replicated and caused caspase-dependent apoptosis in interferon-defective melanoma cells. Virus replication was required for their oncolytic activity. Cisplatin enhanced the oncolytic activity of delNS1 viruses. The cytotoxic drug increased delNS1 replication and delNS1-induced caspase-dependent apoptosis. Interference with MEK/ERK signalling by RNAi-mediated depletion or the MEK inhibitor U0126 did not affect the oncolytic effects of the delNS1 viruses. In oncolysis sensitive melanoma cells, delNS1-IL-15 (but not delNS1) infection resulted in the production of IL-15 levels ranging from 70 to 1140 pg/mL in the cell culture supernatants. The supernatants of delNS1-IL-15-infected (but not of delNS1-infected) melanoma cells induced primary human natural killer cell-mediated lysis of non-infected tumour cells. In conclusion, we constructed a novel oncolytic influenza virus that combines the oncolytic activity of delNS1 viruses with immunostimulatory properties through production of functional IL-15. Moreover, we showed that the oncolytic activity of delNS1 viruses can be enhanced in combination with cytotoxic anti-cancer drugs.

## Introduction

Oncolytic viruses destroy selectively tumour cells sparing non-malignant (normal) cells [1]. During tumorigenesis, cells undergo multiple changes. This results in alterations of the activation status of many different signal transduction pathways, such as the Ras/Raf/MEK/ERK or PI3K/AKT kinase pathways [2] as well as pathways involved in the antiviral response such as interferon signalling. These alterations render tumour cells susceptible to different (engineered) oncolytic viruses while normal cells are not affected by them.

In order to enhance the anti-cancer properties of oncolytic viruses, so called “armed viruses” have been developed. In addition to their oncolytic activities, these viruses encode for various gene products that exert anti-cancer effects. One approach is the introduction of genes encoding for pro-apoptotic proteins or molecules that block constitutively activated oncogenic signalling pathways [1]. Other viruses encode for gene products that exert anti-angiogenic or immunostimmulatory effects [3]–[6].

The influenza A non-structural protein 1 (NS1) is an interferon antagonist. Influenza A viruses in which the NS1 gene has been deleted (delNS1) were shown to specifically lyse interferon-defective cancer cells or cancer cells that express oncogenic Ras [7]–[8]. Furthermore, delNS1 influenza A viruses were shown to induce immune responses through the activation of cytotoxic T-lymphocytes (CTLs) and natural killer (NK) cells [9]–[10]. To enhance the immunostimulatory properties of delNS1 viruses, we developed a novel strategy for the generation of transgenic viruses that stably express cytokines [11]. The influenza A virus genome consists of 8 gene segments. The proteins NS1 and NEP (formerly NS2) are encoded on segment 8. The mRNAs for NS1 or NEP are produced by alternative splicing. We inserted the coding sequence of several cytokines and chemokines (IL-2, IL-24, CCL20) into segment 8 of a delNS1 influenza A virus. The resulting viruses retained their ability to induce the expression of NEP and caused the production of high levels of corresponding chemokines in African Green Monkey kidney Vero cells [11].

Based on this technology, we here generated a novel delNS1 virus encoding for IL-15 (delNS1-IL-15). IL-15 was suggested to be superior and distinct from IL-2 in its potential to generate tumour-specific CTL and NK cell responses [12]–[13]. Recently, an interleukin-15 expressing oncolytic vesicular stomatitis virus showed increased anti-tumor immune response [6]. We chose melanoma as model entity for the investigation of the oncolytic effects and IL-15 expression induced by the delNS1-IL-15 virus. Melanoma cells are known i) to be sensitive to oncolysis by delNS1 virus [7]–[8] and ii) NK-cells as well as T-cells were shown to play crucial roles in the anti-melanoma immune response [14]. The delNS1-IL-15 virus exerted oncolytic effects and induced apoptosis in interferon-defective melanoma cells to a similar extent like the corresponding delNS1 virus. The pro-apoptotic effects of the delNS1 viruses were enhanced in combination with the cytotoxic drug cisplatin. In addition, infection of melanoma cells with delNS1-IL-15 resulted in the production of high levels of biologically active IL-15.

## Materials and Methods

### Cell lines

Colo-679, IPC298, and IGR-39 cell lines were obtained from the DSMZ, Braunschweig, Germany. Mewo cells were received from ECACC, Salisbury, England. All cells were propagated in Iscove's Modified Dulbecco's Medium (IMDM) supplemented with 10% FBS, 100 IU/mL penicillin, and 100 mg/mL streptomycin at 37°C. Cell culture media were purchased from Biochrome (Berlin, Germany).

### Virus preparation

All viruses were generated by reverse genetics as described before [15]. IVR-116 consists of the PB2, PA, NP, NS and M genes from H1N1 A/PuertoRico/8/34; the HA and NA genes from H1N1 A/NewCaledonia/20/99, and the PB1 gene from H3N2 A/Texas/1/77. The delNS1 viruses used in this study consist of the same gene segments as IVR-116 but have a deletion in the NS gene segment (coding for NS1 and NEP by alternative splicing) resulting in the expression of a truncated, non-functional NS1 protein (13 N-terminal amino acids of NS1 left). The interleukin-15 expressing delNS1-IL-15 virus was constructed by methods decribed previously [11]. In brief, the splicing efficiency of NS mRNA was improved by modifying the splice acceptor site. Secretion of the introduced cytokine was achieved by fusing the N-terminal 13 amino acids of NS1 with the partial murine IgK signal peptide, followed by the IL-15 cDNA (Figure S1). All viruses were propagated on Vero (ATCC, CCL-81) cells and cell free virus aliquots were stored at −80°C. Virus titres were determined as 50% tissue culture infectious dose (TCID_50_/mL; 50% tissue culture infectious dose/mL) in confluent Vero cells in 96-well microtitre plates.

### Viability assay

Cell viability was measured by a modified 3-(4, 5-dimethylthiazol-2-yl)-2, 5-diphenyltetrazolium bromide (MTT) dye reduction assay. Briefly, cells grown in 96-well plates were infected with multiplicities of infection (MOIs) ranging from 0.01 to 1. 24, 48, or 72 hours post infection (hpi), MTT-reagent was added and the plates were incubated for 4 hours at 37°C. Subsequently, a 20% SDS solution in 1∶1 DMF∶H_2_O was added and the plates were incubated overnight at 37°C. The absorption was detected at a wavelength of 560 nm and a reference wavelength of 620 nm. The cell viability was calculated relative to non-infected control (mock) cells.

### Immune staining

Melanoma cells grown in 96-well plates were infected at MOI of 1, 0.1 or 0.01 with the indicated viruses. 24 hpi, cells were immuno-stained for influenza A nucleoprotein as described before [16]. Briefly, cells were fixed with ice-cold acetone/methanol (40∶60, Mallinckrodt Baker B.V., Deventer, Netherlands) and stained using the mouse anti-influenza NP monoclonal antibody (Millipore, Schwalbach, Germany). Biotin-conjugated secondary monoclonal antibodies were used and visualisation was performed using a streptavidin-peroxidase complex and 3-amino-9-ethylcarbazole as substrate. The relative numbers of infected cells were expressed in %.

### Virus yield assay

5×10^5^ Colo-679, IPC298, IGR-39, or MeWo cells were infected with delNS1 or delNS1-IL-15 at a MOI of 0.1. At the indicated time points, aliquots of the supernatants were taken and serial 10-fold dilution steps were performed. Virus titres were determined by endpoint dilution titration on Vero cells in 96-well microtitre plates. Plates were incubated for 3–4 days and the wells were visually analysed for virus-induced cytopathogenic effects. Virus titres were calculated as 50% tissue culture infectious dose per millilitre (TCID_50_/mL).

### Determination of the sub-G1 fraction

5×10^5^ Colo-679, IPC298, IGR-39, or MeWo cells were infected with delNS1, delNS1-IL-15 or IVR-116 at MOI 1. 48 hpi, cells were fixed and permeabilised with 70% ethanol overnight at −20°C. The cellular DNA was stained using propidium iodide (20 µg/mL in PBS) and analysed by flow cytometry (FACSCalibur, BD Biosciences, Heidelberg, Germany). Cells with fractional DNA content (“sub-G1” cell subpopulation) are considered to be dead (usually apoptotic) cells. Non-infected cells served as negative controls, cisplatin-treated (10 µg/mL) cells as positive control for apoptosis induction.

### Caspase activation

Caspase activation was measured using the Caspase-Glo 3/7, 8 or 9 assay (Promega, Mannheim, Germany) following the manufacturer's instructions. As control for the induction of apoptosis, cells were treated with 10 µg/mL cisplatin.

### Inhibitors

The caspase-3 inhibitor Z-DEVD-fmk and the pan-caspase inhibitor Z-VAD-fmk were obtained from R&D systems (Minneapolis, USA). To inhibit caspase activation, cells were incubated for 30 minutes with 80 µM of the inhibitors prior to infection.

### Western Blot

For the analysis of the interferon response, 1×10^6^ cells were infected at MOI 5. Preincubation with 5×10^3^ IE/mL Interferon (IFN) β (betaferon, Bayer-Schering Pharma, Berlin, Germany) for 24 hours served as positive control. The cells were lysed 24 hpi in Triton X sample buffer and separated by SDS-PAGE as described before [17]. Proteins were detected using specific antibodies against β-actin (Sigma, München, Germany), STAT1, phospho STAT1 (both Cell Signalling Technology, Danvers, USA), STAT2, phospho STAT2 (both Upstate Cell signalling solutions, New York, Boston, USA), or MxA (Novus Biologicals, Littleton, USA). For the analysis of ERK and AKT activation, 1×10^6^ Colo-679 cells were infected at MOI 1. Protein lysates were taken 24 hpi. Antibodies against ERK1/2, phospho ERK1/2, AKT and phosphpo AKT (all Cell Signalling Technology, Danvers, USA) were used for detection. Visualisation was performed by enhanced chemoluminescene using a commercially available kit (GE Healthcare, Freiburg, Germany).

### RNA interference experiments

The synthetic siRNA oligonucleotides ON-TARGETplus SMARTpool siRNAs targeting ERK1 (encodes ERK1/MAPK3, NM_002746) or ERK2 (encodes ERK2/MAPK1, NM_002745) were purchased from Dharmacon (Lafayette, CO, USA). Non-target siRNA (ON-TARGETplus SMARTpool, Dharmacon) was used as negative control. Colo-679 cells were transfected by electroporation using the NeonTM Transfection System (Invitrogen) according to the manufacturer's protocol. Colo-679 cells were transfected in each case with 2.5 µM siRNA (voltage 1300, width 20, pulses 2). 48 h after transfection, the cells were infected with the investigated viruses.

### Measurement of Interleukin-15 expression (ELISA)

5×10^5^ Colo-679, IPC298, IGR-39, or MeWo cells were infected with delNS1 or delNS1-IL-15 at MOI 1 or mock-infected. Supernatants were collected at the indicated time points. The amounts of IL-15 were determined using the commercially available Human Interleukin-Kit (R&D Systems, Wiesbaden, Germany) according to manufacturer's protocol.

### Measurement of the cytotoxic activity of primary human NK-Cells

5×10^5^ Colo-679 cells were non-infected (mock) or infected with delNS1 or delNS1-IL-15 at MOI 1 and supernatants were collected 24 hours post infection. The cell culture supernatants were UV-inactivated for 20 minutes in order to exclude effects caused by replicating viruses. Primary human NK-cells were isolated from the blood of healthy volunteers as described before [18]. NK-cells were then incubated with the supernatants of infected Colo-679 cells for four days. Thereafter, the NK cell-mediated tumour cell lysis was determined by a coupled luminescent method using the aCella-Tox kit (Cell Technology, Mountain View, CA) as described before [18].

### Statistical Analysis

Two groups were compared by student's t-test, more groups were compared by ANOVA. Pairwise Multiple Comparison was performed by Student-Newman-Keuls-Test.

## Results

### Influence of oncolytic Influenza A viruses on melanoma cell viability

**Figure 1:**
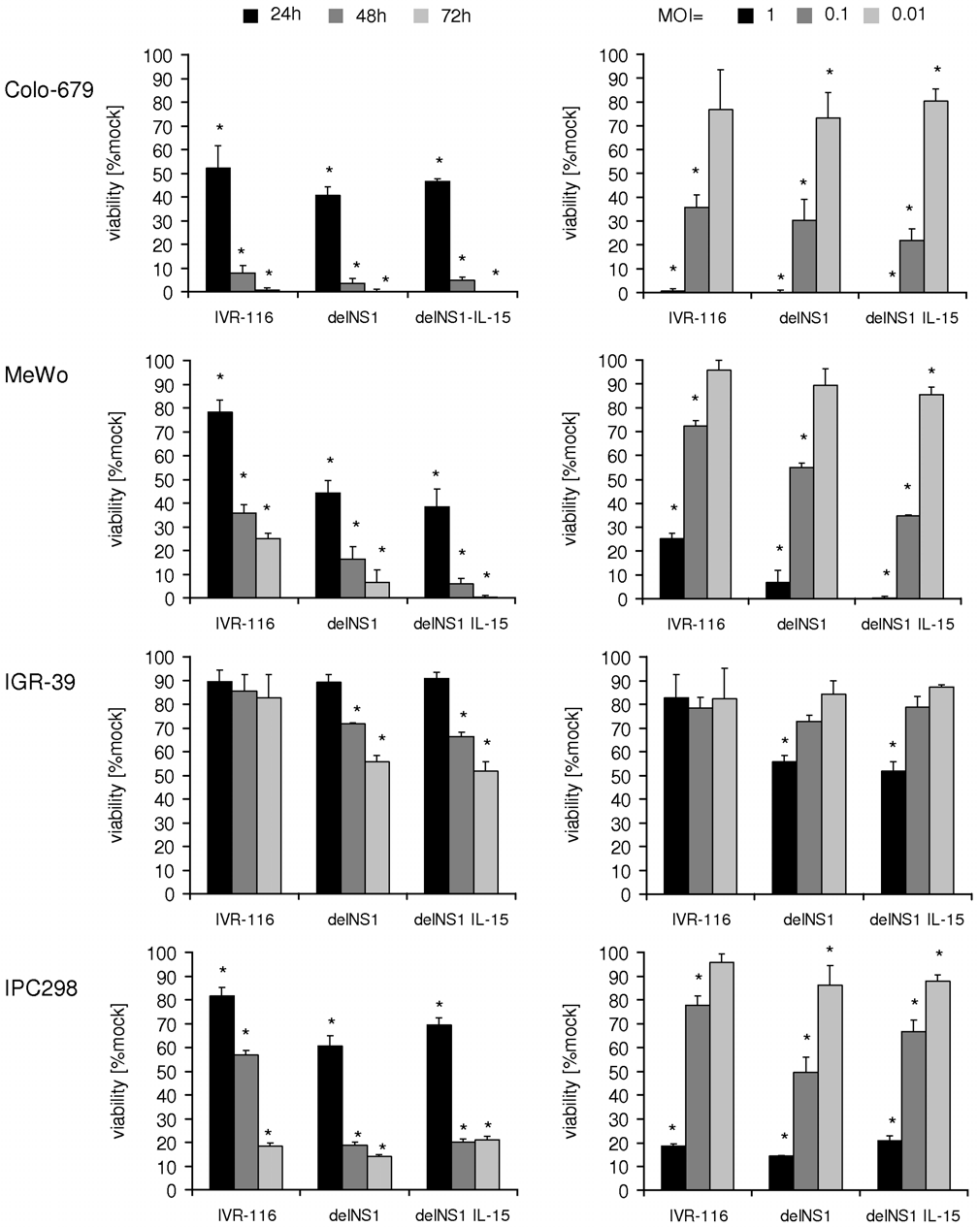
Influence of influenza A virus infection on melanoma cell viability. Cells were infected at a MOI of 1 with IVR-116 (expressing WT NS1), delNS1 or delNS1-IL-15. MTT-assay was performed after 24, 48, and 72 hours post infection (left panel) or cells were infected at MOIs ranging from 1 to 0.01 and MTT-Assay was performed 72 hours post infection (right panel). All data represent mean ± SD of three independent experiments. *significantly different compared to non-infected control (mock), P<0.05.

The IL-15 expressing delNS1-IL-15 influenza A virus was construced by insertion of the human IL-15 coding sequence into the NS gene segment, thereby replacing NS1 (Figure S1). The oncolytic effects of the oncolytic influenza A viruses delNS1, delNS1-IL-15, and the wildtype NS1 expressing IVR-116 virus were examined in four different melanoma cell lines: Colo-679, MeWo, IGR-39, and IPC298. Cells were infected with different MOIs ranging from 0.01 to 1 and the cell viability was assessed 24, 48, or 72 hours post infection (hpi) by MTT-assay. DelNS1 infection reduced the viability of the investigated cell lines in a dose- and time-dependent manner relative to the non-infected control (mock) cells (Figure 1). DelNS1-IL-15 caused similar tumour cell lysis like delNS1. The effects of the delNS1 viruses on cancer cell viability were similar to those of the NS1 wild-type virus IVR-116. All investigated viruses strongly affected the viability of Colo-679, MeWo, and IPC298 cells, but hardly affected IGR-39 cell viability. The maximum oncolytic effect was achieved 72 hpi at MOI 1, when >80% of IPC298 cells and nearly all Colo-679 or MeWo cells were killed. It was shown before that delNS1 Influenza viruses showed no toxic or lytic effects on non-transformed and/or interferon competent cells [7]–[8], [15]. To ascertain that the newly generated delNS1-IL-15 had no effect on non-transformed cells, we infected primary human foreskin fibroblasts (HFF). The viability of HFFs was not affected after infection with delNS1 or delNS1-IL-15 (Figure S2).

### The oncolytic activity of Influenza A viruses depends on virus replication

**Figure 2:**
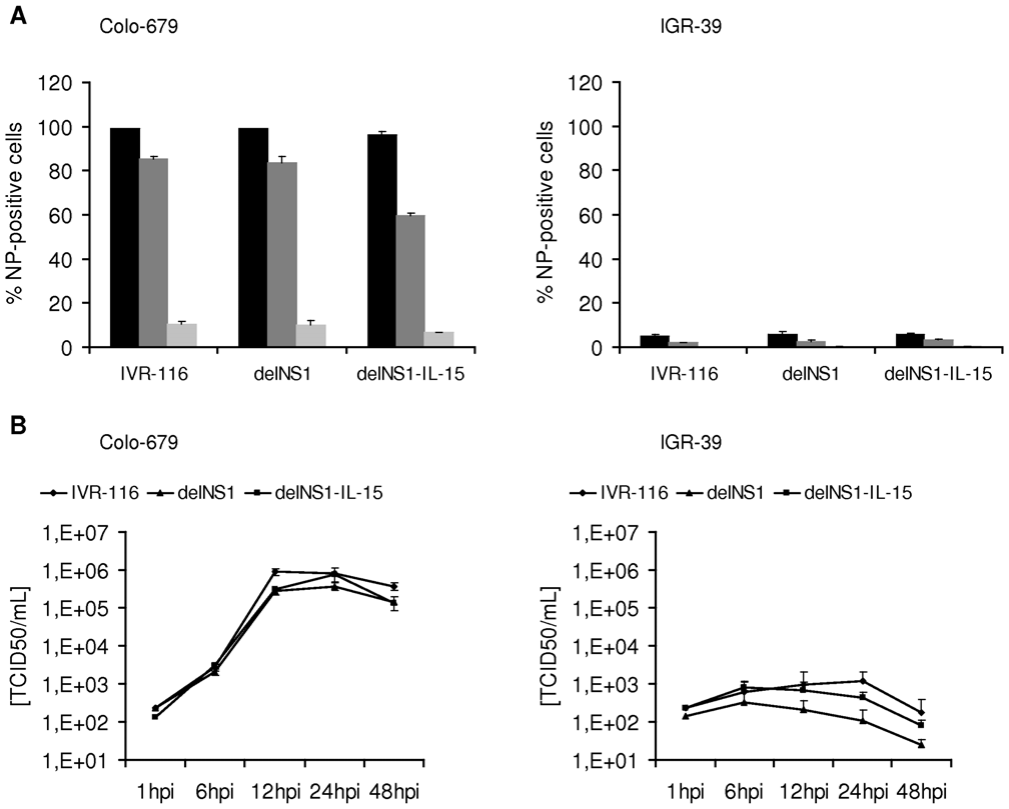
Infection rate and replication kinetics of influenza A viruses. **A**) Immune staining of infected cells. Cells were infected at MOI 1, 0.1 or 0.01 respectively. Staining for influenza A virus NP antigen expression was performed 24 hours post infection. **B**) Cells were infected with IVR-116, delNS1, or delNS1-IL-15 (MOI 0.1). At the given time points, aliquots of the supernatants were taken and the TCID_50_/mL was determined.

To show whether the reduction of melanoma cell viability depends on virus replication, we observed the expression of virus nucleoprotein and the production of progeny infectious virus particles in Colo-679 cells which were sensitive to oncolysis and the non-sensitive cell line IGR-39. 24 hours after infection with delNS1, delNS1-IL-15, or IVR-116 at MOI 1 or 0.1 nearly 100% of Colo-679 cells were infected. At an MOI of 0.01 up to 70% of Colo-679 cells were infected 24 hpi (Figure 2A). As shown in Figure 2B all investigated viruses replicated to titres about 10^6^TCID_50_/mL in Colo-679, reaching the peak after 12 to 24 hours. To further elucidate whether oncolysis depends on virus replication, we infected Colo-679 with UV-inactivated viruses. Infection with UV-inactivated viruses did not influence cell viability (Figure S3). The oncolysis-resistant IGR-39 cells were barely infected (4–6% NP positive cells detectable at MOI 1) and the titres did not exceed 3×10^3^TCID_50_/mL (Figure 2A and 2B).

### DelNS1 viruses induce apoptosis in permissive melanoma cells

**Figure 3:**
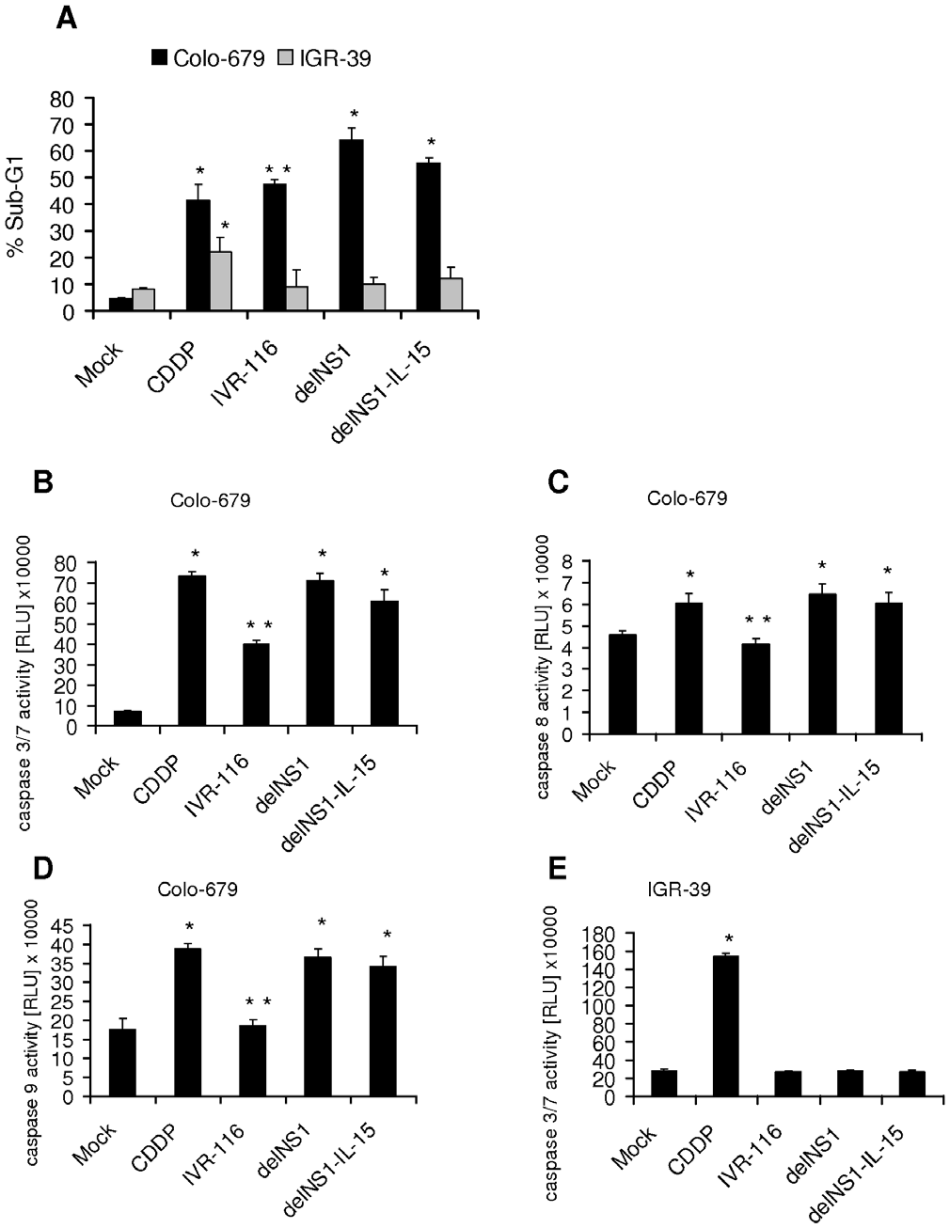
Apoptosis induction in influenza A virus-infected melanoma cells. Cells were infected at MOI 1. 10 µg/mL Cisplatin (CDDP) were used as a control for apoptosis induction. **A**) The percentage of cells in sub-G1-phase was determined 48 hpi. **B–E**) Caspase 3/7, 8, or 9 activity was determined 24 hpi. *significantly different compared to mock, P<0.01, **significantly different compared to delNS1 infection, P<0.01.

**Figure 4:**
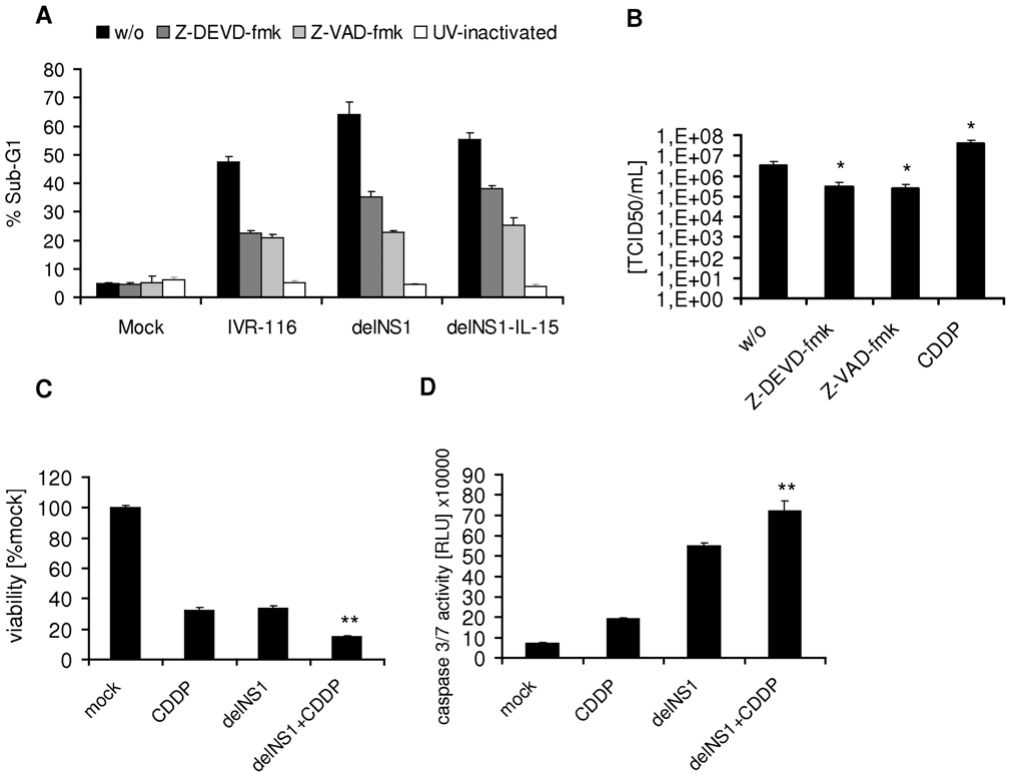
The role of apoptosis in melanoma cell oncolysis. **A**) Determination of Colo-679 cells in sub-G1-phase was performed 48 hpi (MOI 1) by flow cytometry in the absence or presence of the pan-caspase inhibitor Z-VAD-fmk (80 µM) or the caspase 3 inhibitor Z-DEVD-fmk (80 µM). Z-VAD-fmk or Z-DEVD were added to the cell cultures 30 min prior to virus infection. UV-inactivated viruses served as controls. **B**) Influence of Z-VAD-fmk (80 µM), Z-DEVD-fmk (80 µM) or cisplatin (CDDP) on delNS1 replication in Colo-679 cells. Colo-679 cells were treated with the caspase inhibitors 30 min prior to delNS1 (MOI 0.1) infection or treated simultaneously with 0.5 µg/mL cisplatin. Viral titres were determined 24 hpi. **C**) Cells were infected with delNS1 MOI 0.1 and treated with 0.5 µg/mL CDDP. Cell viability was determined 48 hpi by MTT-assay and **D**) Caspase 3/7 was measured 24 hpi. w/o = without treatment. *significantly different compared to non treated P<0.05; **significantly different than delNS1-infection and CDDP-treatment alone P<0.05.

Influenza A viruses (avian H5N1 as well as H1N1) are known to induce caspase dependent apoptosis in different cell types [16], [19]–[20]. The induction of caspase 3 during influenza virus infection is known to be important both for virus induced cell death and virus replication [19]. To study the induction of cell death and the role of caspases in melanoma cell oncolysis by delNS1 and delNS1-IL-15, we determined the fraction of sub-G1 cells by flow cytometry and measured the activation of caspase 3/7, 8 or 9 after virus infection. DelNS1 and delNS1-IL-15 increased the numbers of sub-G1 cells by up to 67% in comparison to non-infected cells in Colo-679 (Figure 3A). The activities of caspase 3/7, caspase 8, and caspase 9 were significantly induced in delNS1- and delNS1-IL-15-infected Colo-679 cells (Figure 3B–D). Compared to the delNS1 viruses, IVR-116 induced lower levels of sub-G1 cells and less caspase 3/7, 8 and 9 activitation. The infection of IGR-39 with IVR-116, delNS1, or delNS1-IL-15 did neither alter the number of cells in sub-G1 phase, nor induce caspase 3/7 activity (Figure 3A and 3E). The caspase 3 inhibitor Z-DEVD-fmk and the pan-caspase inhibitor Z-VAD-fmk were used to investigate to which extent the induction of cell death depends on caspase activation. Both caspase inhibitors reduced the numbers of sub-G1 cells in influenza A virus-infected Colo-679 cell cultures (Figure 4A). Also, both caspase inhibitors reduced the virus titres produced by delNS1-infected Colo-679 cells (Figure 4B). To examine whether the virus-induced apoptosis depends on viral replication, we used UV-inactivated viruses. Infection of Colo-679 cells with inactivated IVR-116, delNS1, or delNS1-IL-15 viruses did neither affect the sub-G1 cell numbers (Figure 4A) nor caspase 3/7 activation (data not shown). To investigate the influence of an apoptosis inducing cytotoxic drug on oncolysis we combined cisplatin with delNS1 infection. The combination of 0.5 µg/mL cisplatin and delNS1 caused significantly increased oncolytic effects in comparison to either single treatment alone (Figure 4C). Additionally, cisplatin enhanced the delNS1-induced caspase3/7 activation and the delNS1 virus production in Colo-679 cells significantly (Figure 4D and 4B).

### DelNS1 virus causes oncolysis in interferon defective cells

**Figure 5:**
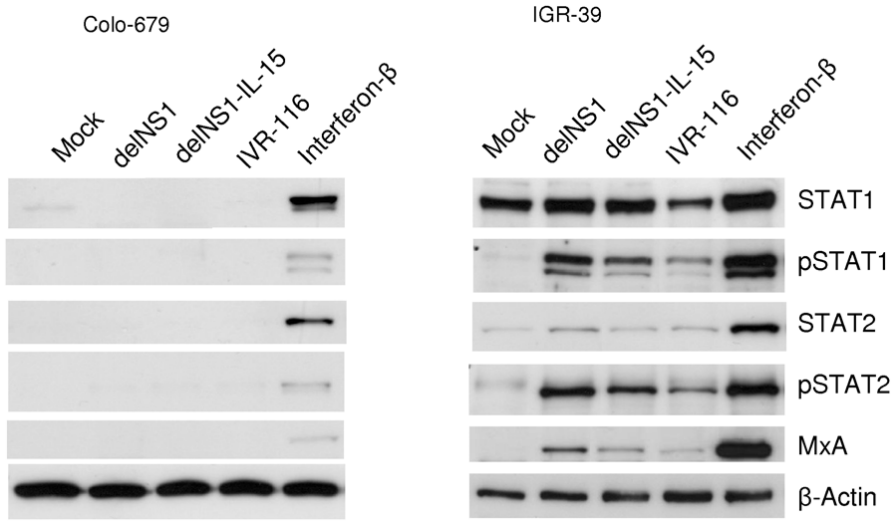
Induction of interferon response in delNS1-infected melanoma cells. Colo-679 cells and IGR-39 cells were infected with IVR-116, delNS1 or delNS1-IL-15 respectively (MOI 5). Cells treated with 5000 IU/mL Interferon-β served as control for the induction of interferon response. Protein levels of STAT1, phosphorylated STAT1 (pSTAT1), STAT2, phosphorylated STAT2 (pSTAT2), MxA, and β-Actin were determined 24 hpi by Western blot.

DelNS1 viruses were shown to replicate in cancer cells with impaired interferon response, but not in interferon competent cells [8], [15]. Thus, we compared the induction of the interferon response in highly delNS1-permissive (oncolysis-sensitive) Colo-679 cells and the low-permissive (oncolysis-resistant) IGR-39 cells. MxA expression is induced by type I and III interferons through STAT1/STAT2 signalling, but not directly by virus components or RNA-intermediates [21]. The induction of MxA expression can therefore be used as a surrogate marker for interferon response. The infection of Colo-679 cells with delNS1 or delNS1-IL15 did not induce STAT1 and STAT2 phosphorylation or MxA expression indicating a lack of interferon signalling (Figure 5). The infection of IGR-39 cells with delNS1 or delNS1-IL-15, however, resulted in STAT1 and STAT2 phosphorylation and upregulation of MxA expression indicating a cellular interferon response. In comparison to the delNS1 viruses, the IGR-39 cell interferon response was reduced after infection with wildtype NS1 expressing IVR-116 virus as indicated by decreased levels of phosphorylated STAT1 and STAT2 and alleviated MxA expression.

### ERK1/2 activation is not crucial for melanoma cell oncolysis

**Figure 6:**
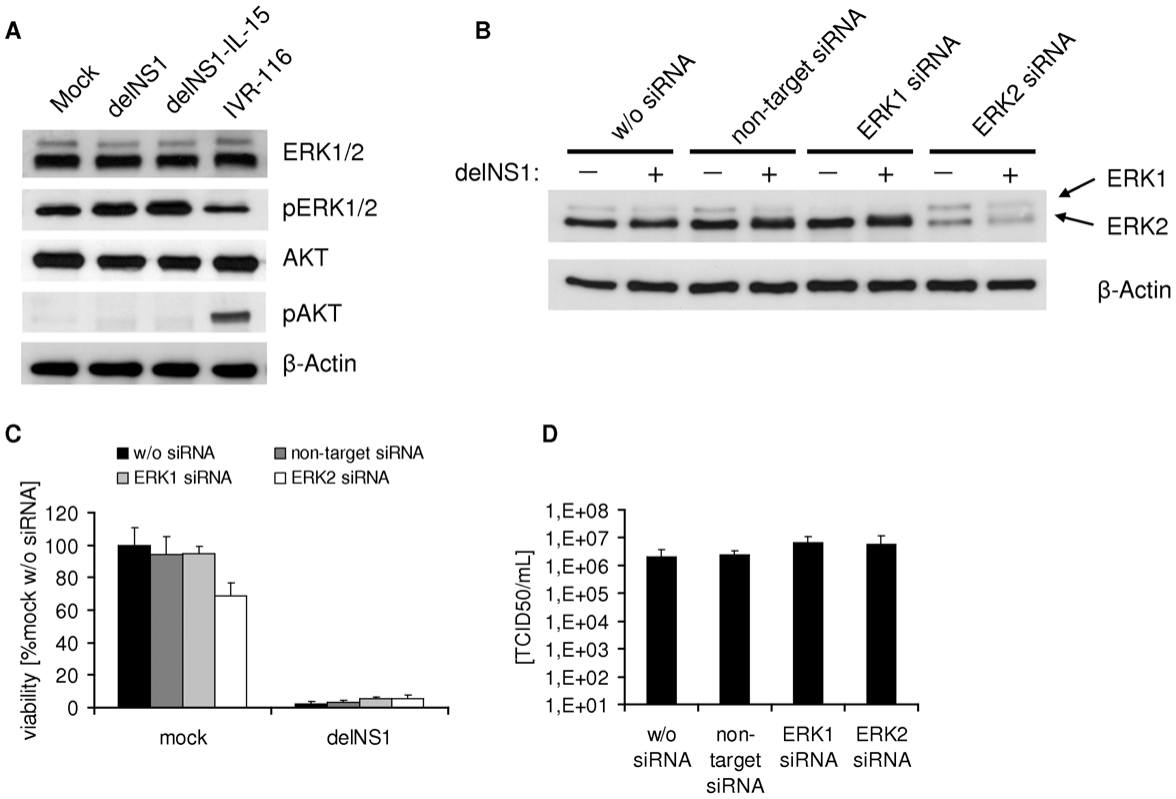
Cell signaling pathways activated by oncolytic influenza A viruses in melanoma cells. **A**) Colo-679 cells were infected with IVR-116, delNS1 or delNS1-IL-15 respectively (MOI 1). Protein levels of ERK1/2, phosphorylated ERK1/2 (pERK1/2), AKT, phosphorylated AKT (pAKT) and β-Actin were determined 24 hpi by Western blot. **B–D**) The role of ERK1/2 phosphorylation in melanoma cell oncolysis by delNS1. Colo-679 cells were transfected with siRNA directed against ERK1, ERK2, or non-target siRNA and infected at MOI 1 with delNS1 48 hpi. **B**) Western blot analysis of ERK1 and ERK2 levels. Western blot extracts were taken 24 hpi. **C**) The cell viability was assesed 48 hpi by MTT assay. **D**) Virus titres were determined as TCID_50_/mL 24 hpi.

Signal transduction pathways like the Ras/Raf/MEK/ERK signalling pathway or the PI3K/AKT signalling pathway may be stimulated by Influenza A infection and may play a role in virus replication and tumour cell survival [2], [20], [22]. First, we determined whether ERK1/2 and/or AKT were activated by delNS1 viruses in Colo-679 cells. Non-infected Colo-679 cells showed constitutive activation/phosphorylation of ERK1/2, but not of AKT (Figure 6A). Infection of Colo-679 cells with delNS1 or delNS1-IL-15 viruses did not influence AKT phosphorylation, but resulted in increased ERK1/2 phosphorylation. In contrast, infection with the wild-type NS1 expressing IVR-116 virus appeared to suppress ERK1/2 phosphorylation and to increase AKT phosphorylation. To investigate whether activation of ERK1/2 may be crucial for efficient delNS1 virus-mediated oncolysis, we performed knock-down experiments with siRNA directed against ERK1 or ERK2. The analysis of cellular ERK levels indicated effective down-regulation of ERK1 or ERK2 48 h after siRNA transfection (Figure S4). Thus, we infected Colo-679 48 h after the transfection procedure with delNS1 (MOI 1). As shown in Figure 6B the levels of ERK1 or ERK2 remained diminished during the 24 h of the delNS1 infection experiment in Colo-679 cells. However, the down-regulation of ERK1 or ERK2 did not affect the delNS1-induced oncolytic effects or delNS1 replication (Figure 6C and 6D). In accordance, U0126, an inhibitor of the ERK 1/2 up-stream kinases MEK 1/2, did not impair the oncolytic effects of delNS1 (Figure S5).

### DelNS1-IL-15-infected melanoma cells produce biologically active interleukin-15

**Figure 7:**
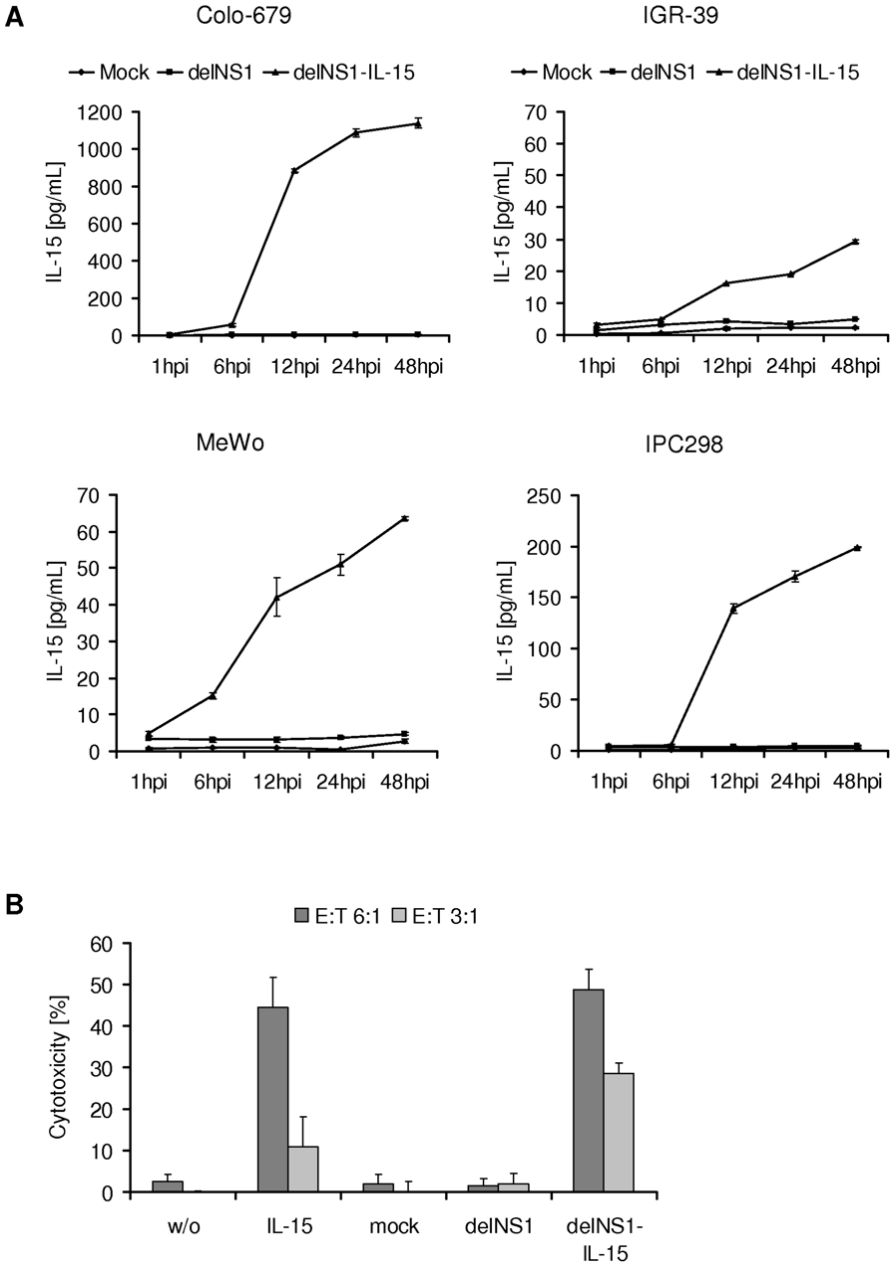
Expression of biological active interleukin-15. **A**) The cell lines were infected with delNS1 or delNS1-IL-15 at MOI 1 or non-infected (mock). Supernatants were analysed for interleukin-15 by ELISA after the indicated incubation periods. **B**) Induction of natural killer (NK) cell mediated tumour cell lysis. Primary human NK-cells were incubated with cell culture supernatants of mock, delNS1-, or delNS1-IL-15-infected Colo-679 cells. After four days of incubation, NK-cells were analysed for cytotoxicity against non-infected tumour target cells. NK-cells incubated without or with 100 U recombinant IL-15 served as control.

To examine whether delNS1-IL-15-infected melanoma cells produce and secrete IL-15, the amount of IL-15 was measured in the cell culture supernatants of infected cells by ELISA. As shown in Figure 7A, IL-15 concentrations of 1140 pg/mL (Colo-679), 70 pg/mL (MeWo), and 200 pg/mL (IPC298) were detected in the supernatants of delNS1-IL-15-infected melanoma cells. The maximal IL-15 concentration detected in non-permissive IGR-39 cells was 30 pg/mL. The supernatants of mock- or delNS1-infected cells did not contain measurable IL-15 levels.

IL-15 can activate T-cells as well as NK cells [13]. To investigate whether IL-15 expressed by delNS1-IL-15-infected cells is functional, the supernatant of delNS1-IL-15-infected Colo-679 cells was examined for its ability to stimulate NK-cells. Freshly isolated primary human NK-cells were incubated for four days with cell culture supernatants of non-infected (mock), delNS1-, or delNS1-Il-15-infected Colo-679 cells. NK-cells incubated with supernatants of delNS1-IL-15-infected Colo-679 cells displayed a similar target cell killing efficiency (49% cytotoxicity) like NK cells stimulated with 100 U of recombinant IL-15 (45% cytotoxicity) (Figure 7B). In contrast, NK-cells incubated with supernatants of non-infected (mock) or delNS1-infected cells did not induce NK-cell-mediated cytotoxicity.

## Discussion

Influenza A viruses with complete or partial deletions of the NS gene segment showed oncolytic activity in cultures and/or animal models of melanoma, prostate carcinoma, and colon carcinoma [7]–[9], [23]. Here, we investigated the anti-tumour activity of delNS1-IL-15 influenza A viruses, encoding IL-15 from the NS gene segment, in comparison to the corresponding delNS1 virus in a panel of human melanoma cell lines. DelNS1-IL-15 virus replicated in and induced death of interferon-defective melanoma cells to a similar extent like the non-modified delNS1 virus. These results demonstrate for the first time that the expression of foreign genes from the genome of oncolytic delNS1 viruses does not interfere with their antitumoural activity.

Influenza A viruses may induce both caspase-dependent or caspase-independent apoptosis in different cell types including those derived from different tumor entities [19], [24]. The inhibition of influenza A virus-induced caspase 3 activity prevented virus-induced cell death and decreased virus replication [19]. In concordance, we found that caspase 3 is activated during delNS1 virus replication in melanoma cells. Furthermore delNS1 viruses induced activation of caspase 9 and to a minor extent caspase 8, suggesting that both, intrinsic and extrinsic apoptotic pathways are triggered in infected melanoma cells. Notably, the cytotoxic anti-cancer drug cisplatin enhanced the delNS1 virus-induced apoptosis, the delNS1-induced oncolytic effects, and delNS1 virus replication in melanoma cells. These data support the further investigation of the anti-cancer effects of delNS1 viruses in combination with drugs that cause cancer cell apoptosis. A specific inhibitor of caspase 3 as well as a pan-caspase inhibitor suppressed virus replication and partially prevented melanoma cells from virus-induced cell death. The failure of both caspase inhibitors to completely suppress virus-induced cell death suggests that mechanisms other than caspase-dependent apoptosis may be involved in delNS1 virus-induced cell death. In this context, several wild type influenza A virus strains were shown to induce necrosis in a human colon carcinoma cell line while they caused apoptosis in non-malignant cells [24].

There is evidence that the NS1 protein of influenza A viruses may act as inhibitor [25]–[27] or inducer [28]–[29] of cellular apoptosis. Among other mechanisms, the anti-apoptotic effects of NS1 were attributed to the stimulation of the cellular PI3K/AKT survival pathway [16], [30]. The NS1 protein was shown to activate PI3K/AKT signalling by directly interacting with the p85 subunit of PI3K [22], [31]. In the investigated melanoma cells, delNS1 showed a stronger apoptosis induction than the corresponding IVR-116 virus expressing wild-type NS1 protein. This may be due to the decreased activation of the PI3K/AKT pathway by the delNS1 viruses, since AKT was phosphorylated in IVR-116-infected cells but not in delNS1-infected cells. Remarkably, PI3K/AKT activation has been associated with tumour progression and increased response to antitumoral therapy in malignant cells including melanoma cells [2].

Activation of the Ras/Raf/MEK/ERK signalling pathway during influenza infection seems to be important for the succesful replication of influenza A viruses in host cells [20]. A specific blockade of the Ras/Raf/MEK/ERK strongly impaired the growth of different influenza A strains both in vitro and in animal models [20], [32]. It was also shown that Ras/Raf/MEK/ERK signalling may be involved in oncolysis caused by an influenza A virus with a partially deleted (truncated) NS gene (TR-NS1 virus) [9]. The infection of the human prostate cancer cell line LNCaP with TR-NS1 resulted in ERK1 activation. Since U0126, an inhibitor of the ERK up-stream kinases MEK 1/2, inhibited the TR-NS1-induced oncolytic effects this ERK1 phosphorylation was suggested to be critical for the oncolytic effects of TR-NS1 in this cell line. However, our results do not provide evidence that Ras/Raf/MEK/ERK signalling may be of relevance in the context of the delNS1-mediated oncolytic activity in the investigated melanoma cells. U0126 treatment did not interfere with the delNS1-induced oncolytic effects in our experiments. In addition, siRNA-mediated depletion of ERK1 or ERK2 did not impair delNS1 replication or delNS1-induced tumour cell lysis. Among the investigated melanoma cell lines, three display constitutive activation of the Ras/Raf/MEK/ERK pathway through activating V600E BRAF mutation or NRAS mutations: Colo-679 (BRAF V600E mut; Nras wt), IGR-39 (BRAF V600E mut; Nras wt), IPC298 (Braf wt; Nras Q61K mut) [33]–[35]. The BRAF and NRAS wild-type cell line MeWo lacks constitutive activation of Ras/Raf/MEK/ERK signalling [33], [36]. However, there were no major differences in the sensitivities to delNS1 virus-mediated oncolysis between cells with constitutively activated Ras/Raf/MEK/ERK pathway (Colo-679, IPC298) and cells without deregulated Ras/Raf/MEK/ERK pathway (MeWo). In addition, V600E Braf mutated IGR-39 cells with constitutively activated Ras/Raf/MEK/ERK pathway, were insensitive to delNS1-mediated oncolysis.

Further experiments revealed that delNS1 viruses induced a strong interferon response in delNS1-insensitive IGR-39 cells but failed to induce an interferon response in delNS1-sensitive Colo-679 cells. These findings suggest that a defect in the interferon signalling may be relevant for the oncolytic activity of delNS1 viruses in the investigated melanoma cells. However, although the NS1-expressing IVR-116 virus caused a substantially weaker interferon response in IGR-39 cells its replication was nevertheless very limited in this cell line. In this context, low number of IGR-39 cells expressed influenza A virus NP antigen, after infection at MOI 1. This suggests that the virus infection may be restricted at early stages of virus replication cycle, such as adsorption and/or virus uncoating. Thus, also other mechanisms than a lack of interferon response may play a role in the sensitivity of cancer cells to oncolytic influenza A viruses.

Recently, we developed a method for the generation of delNS1 vectors that express foreign proteins from the NS gene segment by the modulation of the splicing efficiency and enhancement of the expression levels of secreted protein by fusing the N-terminal 13 amino acids of NS1 with an IgK-derived secretion signal peptide [11]. DelNS1 viruses armed with genes encoding for cytokines including IL-2 and IL-24 as well as chemokine CCL20 were shown to replicate in the African green monkey kidney cell line Vero and to produce the respective proteins [11]. In the present study, we used this approach for an IL-15 expressing delNS1 vector and showed that this delNS1-IL-15 virus replicates and produces IL-15 in permissive human melanoma cells. The IL-15 produced by delNS1-IL-15-infected cancer cells was biologically active. The supernatants of delNS1-IL-15- (but not of delNS1-) infected cells stimulated primary NK cell lysis of non-infected target cells.

In conclusion, the present study describes the generation of an IL-15-armed oncolytic delNS1 virus and delineates the molecular mechanisms by which delNS1 viruses including delNS1-IL-15 exert oncolytic effects in human melanoma cells. The oncolytic effects of delNS1 viruses are enhanced in combination with the cytotoxic anti-cancer drug cisplatin. In addition, we show for the first time that a delNS1 virus encoding for a human cytokine induces the production of a biologically active cytokine in human tumour cells. These results warrant the further investigation of delNS1-IL-15 as oncolytic influenza A virus that combine oncolytic with enhanced immunomostimulatory properties alone and in combination with cytotoxic drugs.

## Supporting Information

Figure S1 **Schematic illustration of the influenza A NS gene segment.** The wildtype (wt) NS gene segment (of IVR-116) is shown in the upper part. The two transcripts (NS1 and NS2/NEP) are shown. In the lower part the NS1 transcript of delNS1-IL-15 is shown. SAS, splice acceptor site; SDS, splice donor site; bp/py, branch point sequence plus 20-nucleotide pyrimidine stretch; IgK-SP, partial mouse IgKappa signal peptide.
(TIF)

Figure S2 **Reduction of cell viability of human foreskin fibroblasts.** Human foreskin fibroblasts (HFF) were infected with indicated viruses at MOI 1 and cell viability was assessed 72 hpi by MTT assay. Data represent means ± SD of triplicates of a representative experiment.
(TIF)

Figure S3 **Reduction of cell viability by UV-inactivated Influenza A viruses.** Colo-679 were infected with indicated UV-inactivated viruses at MOI 1 and cell viability assay (MTT-assay) was performed 48 hpi. Data represent means ± SD of triplicates of a representative experiment.
(TIF)

Figure S4 **Efficiency of transfection with siRNAs against ERK1 and ERK2.** Protein extracts were taken 48 hours after transfection with indicated siRNA and analysed for ERK1 and ERK2 levels by Western blot.
(TIF)

Figure S5 **Influence of MEK1/2 inhibitor U0126 and delNS1 on cell viability.** Colo-679 cells were infected with delNS1 at a MOI of 0.1 in the absence or presence of the MEK 1/2 inhibitor U0126 (20 µM or 10 µM U0126). The cell viability was determined by MTT assay 48 hours post infection.
(TIF)
