# Supplementary figures and images for: Oncolytic Effects of a Novel Influenza A Virus Expressing Interleukin-15 from the NS Reading Frame
Source: PLoS One. 2012 May 1;7(5):e36506. doi: 10.1371/journal.pone.0036506 (PMC3341362; doi:10.1371/journal.pone.0036506)

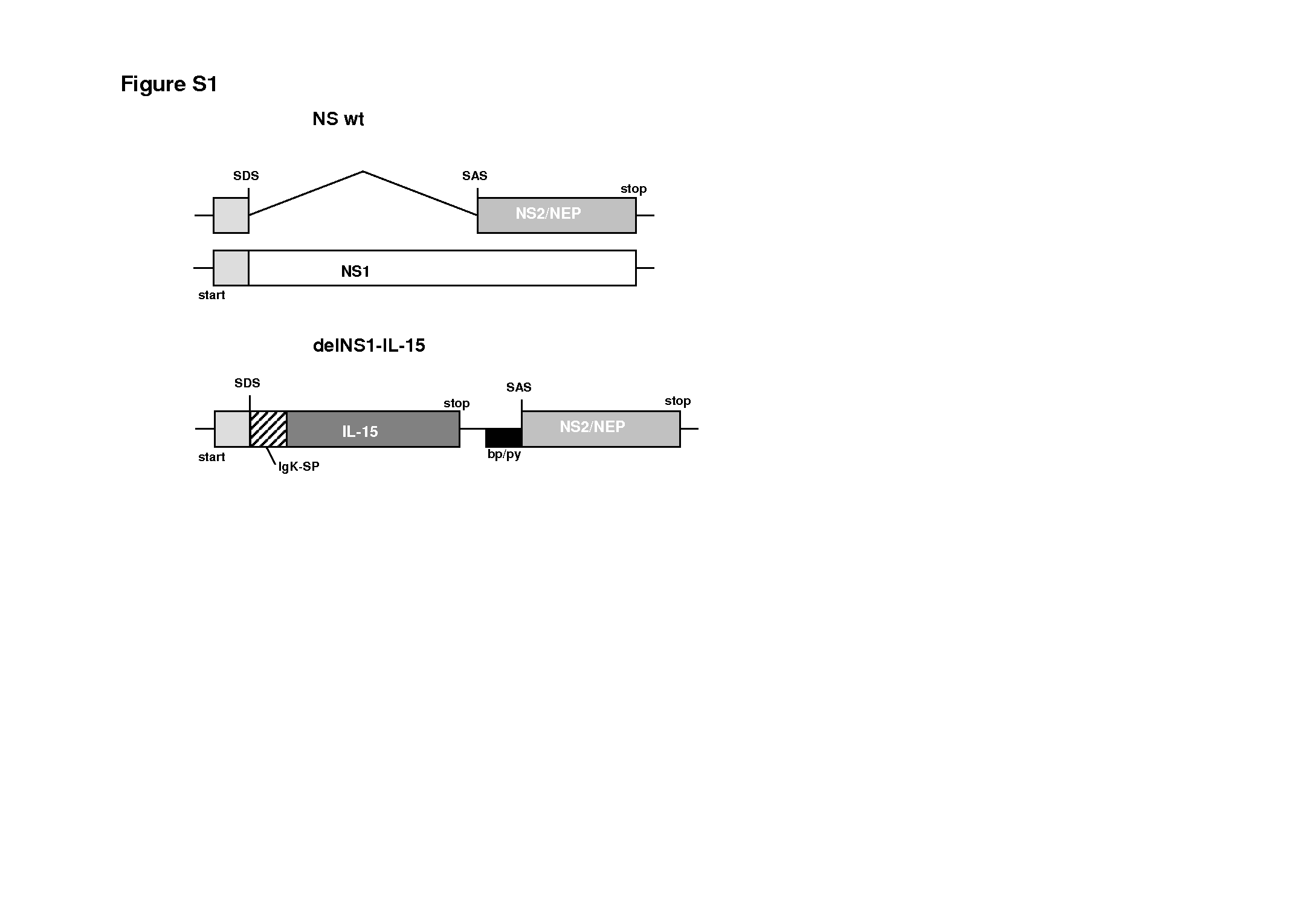

Supplement: Figure S1 — Schematic illustration of the influenza A NS gene segment. The wildtype (wt) NS gene segment (of IVR-116) is shown in the upper part. The two transcripts (NS1 and NS2/NEP) are shown. In the lower part the NS1 transcript of delNS1-IL-15 is shown. SAS, splice acceptor site; SDS, splice donor site; bp/py, branch point sequence plus 20-nucleotide pyrimidine stretch; IgK-SP, partial mouse IgKappa signal peptide. (TIF) [file pone.0036506.s001.tif]
